# Chemically defined and xenogeneic-free differentiation of human pluripotent stem cells into definitive endoderm in 3D culture

**DOI:** 10.1038/s41598-018-37650-z

**Published:** 2019-01-30

**Authors:** Ulf Diekmann, Hanna Wolling, Rabea Dettmer, Isabell Niwolik, Ortwin Naujok, Falk F. R. Buettner

**Affiliations:** 1 Institute of Clinical Biochemistry, Hannover Medical School, Hannover, Germany; 2 REBIRTH Cluster of Excellence, Hannover Medical School, Hannover, Germany

## Abstract

*In vitro* differentiation of human pluripotent stem cells (hPSCs) into definitive endoderm (DE) represents a key step towards somatic cells of lung, liver and pancreas. For future clinical applications, mass production of differentiated cells at chemically defined conditions and free of xenogeneic substances is envisioned. In this study we adapted our previously published two-dimensional (2D) DE induction protocol to three-dimensional (3D) static suspension culture in the absence of the xenogeneic extracellular matrix Matrigel. Next, fetal calf serum and bovine serum albumin present in the standard medium were replaced by a custom-made and xeno-free B-27. This yielded in a chemically defined and xenogeneic-free 3D culture protocol for differentiation of hPSCs into DE at efficiencies similar to standard 2D conditions. This novel protocol successfully worked with different hPSC lines including hESCs and hiPSCs maintained in two different stem cell media prior to differentiation. DE cells obtained by our novel BSA-free 3D protocol could be further differentiated into PDX1- or NKX6.1-expressing pancreatic progenitor cells. Notably, upon DE differentiation, we also identified a CXCR4^+^/NCAM^+^/EpCAM^low^ cell population with reduced DE marker gene expression. These CXCR4^+^/NCAM^+^/EpCAM^low^ cells emerge as a result of Wnt/beta-catenin hyperactivation via elevated CHIR-99021 concentrations and likely represent misspecified DE.

## Introduction

Human pluripotent stem cells (hPSCs) possess an unlimited proliferative potential and can be differentiated into all somatic cell types. Owing to these properties they represent an attractive cell source for cell replacement therapies, pharmacological studies on defined somatic cell types and basic research such as the study of human development^1^.

*In vitro*, hPSCs can be specifically differentiated towards the definitive endoderm (DE) in a process recapitulating gastrulation^2^ providing the basis for further specification towards endoderm-derived cell types^3^. Numerous protocols have been published to differentiate hPSCs into DE cells that differ in composition, concentration and timed application of growth factors (mostly activin A, BMP4, Wnt3A or bFGF) and/or small molecules (mostly glycogen synthase kinase 3 [GSK3] inhibitors [CHIR or BIO] and phosphatidyl-inositol-3-kinase [PI3K] inhibitors^4–13^. Commonly, DE differentiation of hPSCs is induced by contemporaneous activation of Wnt/beta-catenin and activin A signaling for one day followed by continuous activation of activin A signaling alone^4,8,14^. Alternative to Wnt pathway activation, induction of BMP signaling by BMP4 was found to synergistically act with activin A to induce DE formation^9,10,15^. The efficiency of DE formation in the presence of activin A is higher at lower levels of serum supplementation^8^. Serum contains insulin-like growth factor (IGF) which inhibits DE differentiation of hPSCs by elevated PI3K signaling^12^. However, PI3K signaling is required for self-renewal of hPSCs and the removal of serum at the beginning of DE differentiation in combination with activin A is associated with massive cell death^13,16,17^. Thus, to improve cell survival, DE differentiation media commonly contain the xenogeneic supplements fetal calf serum (FCS), bovine serum albumin (BSA) or B-27^TM^ ^16,18–20^. B-27^TM^ is a serum-free media supplement composed of vitamins, antioxidants, lipids, steroids and trace elements but it also contains BSA^21^. DE differentiation is commonly initiated from two-dimensional (2D) cultures of adherent cellular monolayers on xenogeneic matrices like Matrigel^4,14^ or on murine embryonic fibroblasts^8^. Alternatively, hPSCs can be differentiated towards the DE in three-dimensional (3D) culture as cellular aggregates embedded in extracellular scaffolds, which, however, are often based on Matrigel^15,22,23^. In a different approach efficient DE differentiation was achieved from singularized hPSCs that initially formed spheroids in suspension culture without addition of exogenous extracellular matrices but the differentiation was performed in the presence of xenogeneic BSA^24^. A further advantage of culturing hPSCs in 3D is the possibility for scalable expansion in stirred bioreactors enabling the mass production of differentiated cells as desired for medical applications^25,26^.

To the best of our knowledge, no chemically defined and xenogeneic-free approach for DE differentiation of hPSCs as free-floating suspension culture spheroids in 3D culture has been published until today. Thus, we established static 3D conditions for DE differentiation of hPSCs in the absence of xenogeneic scaffolds and media supplements based on our previously published protocol^4^. All xenogeneic components were successively replaced or removed without negative effects compared to the original protocol. 3D conditions also supported further differentiation of DE cells towards PDX1-positive pancreatic progenitors (PPs)^27^ using an adapted, chemically defined and BSA-free media formulation. Additionally, we noticed that DE differentiation conditions gave rise to a subpopulation of CXCR4^+^ that was also positive for NCAM. These cells could be associated with a decreased expression of important DE marker genes suggesting that DE differentiation protocols should be optimized towards low NCAM-positivity.

## Results

### hPSCs can be efficiently differentiated into the DE under 3D culture conditions

**Figure 1:**
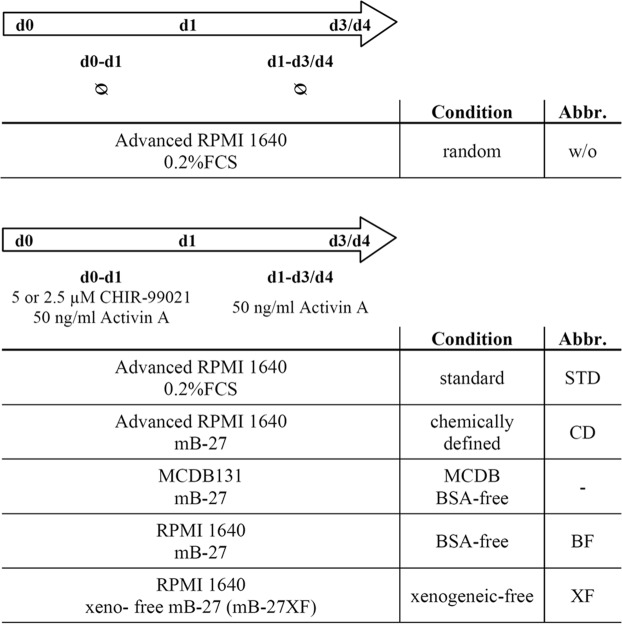
Overview of media used for DE differentiation of hPSCs. DE differentiation of hPSCs was performed according to the CA-A approach^4^, but with different basal media and supplements. Media formulations were changed step-wise. First, FCS was replaced by a modified B-27 (mB-27) to obtain chemically defined conditions. Second, advanced RPMI 1640 was replaced by MCDB131 or RPMI 1640 for BSA-free conditions. Third, mB-27 was replaced by mB-27XF to obtain a fully xenogeneic-free condition.

**Figure 2:**
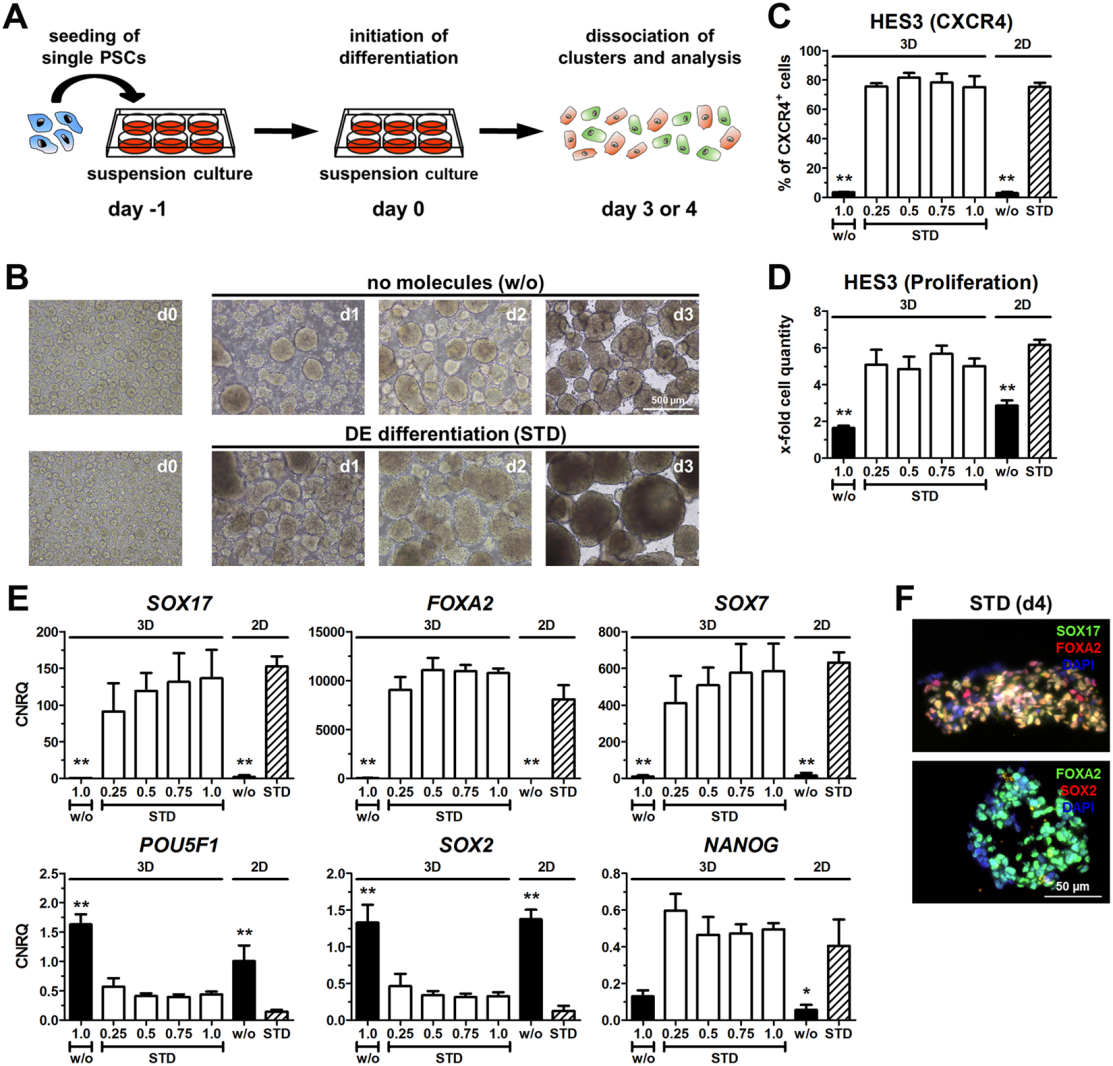
Differentiation towards DE in 2D and 3D. (**A**) Experimental setup for differentiation in 3D culture. (**B**) Representative light microscopic images of HES3 cell clusters in 3D suspension culture at different days during randomized (w/o) or standard (STD) differentiation. Scale bar: 500 µm. (**C**–**E**) Differentiation of HES3 in 3D or 2D under randomized conditions (w/o, inoculum of 1.0 × 10^6^ cells) or under standard conditions (STD, inoculum 0.25 to 1.0 × 10^6^ cells) using 5 µM CHIR. Values are means ± SEM, n = 4–8. Statistical analysis was performed with ANOVA plus *Dunnett’s* post hoc test, *p < 0.05, **p < 0.01 compared to the STD-2D condition (striped column). (**C**) Quantification of CXCR4^+^ cells by flow cytometry. (**D**) Cell proliferation in relation to inoculated cell number. (**E**) Normalized expression of marker genes for DE (*SOX17*, *FOXA2*), extra-embryonic endoderm (*SOX7*) and pluripotency (*NANOG*, *POU5F1*, *SOX2*) scaled to undifferentiated cells (CNRQ = calibrated normalized quantities). (**F**) Representative fluorescence micrographs of HES3 at day 4 of STD-3D differentiation. Depicted are SOX17 (green) and FOXA2 (red) in the upper image and FOXA2 (green) and SOX2 (red) in the lower image. Nuclei were counterstained with DAPI (blue). Scale bar: 50 µm.

An overview of the tested culture conditions and their abbreviations is presented in Fig. 1. Our previously published adherent DE differentiation protocol relies on media supplementation with CHIR-99021 (CHIR) and activin A for one day and with activin A on the consecutive days (CA-A protocol^4^), which will be herein referred to as standard (STD)-2D. This protocol was adapted to a small-scale static suspension culture (STD-3D) to exclude Matrigel (Fig. 2A). Dissociated human embryonic stem cells (hESCs) of the cell line HES3 were cultured in suspension for 24 h until they formed small clusters. Subsequently, randomized differentiation (w/o) or DE differentiation according to the STD-3D approach was induced. The STD-3D protocol resulted in larger and more densely packed clusters compared to the randomized condition (Fig. 2B). Different cell densities (0.25–1.0 × 10^6^ hPSCs inoculated per six well) were tested for the STD-3D culture condition and yielded similar percentages of CXCR4^+^ cells and rates of proliferation comparable with the STD-2D condition (Fig. 2C,D). Thus, by increasing the inoculum, higher numbers of cells committed to the DE are yielded. As expected, randomized differentiation (w/o) in 2D or 3D culture did not induce significant numbers of CXCR4^+^ cells (Fig. 2C). Expression analysis of DE marker genes (*SOX17*, *FOXA2*) revealed strong inductions only after STD differentiation at all tested cell densities in 3D culture, which were similar to the STD-2D condition. *SOX7* gene expression was also comparable between STD-3D and STD-2D conditions, which excluded an extensive differentiation into extra-embryonic endoderm in 3D culture. Pluripotency markers (*POU5F1*, *SOX2*, *NANOG*) showed similar expression levels after four days of differentiation comparing 3D with 2D STD conditions (Fig. 2E). HES3 differentiated in STD-3D showed a distinct co-localization of SOX17 and FOXA2 proteins as a hallmark of proper DE development and FOXA2-positive cells were negative for SOX2 (Fig. 2F). Taken together, the STD-2D protocol for DE differentiation^4^ of hPSCs could be adapted to static suspension culture (STD-3D) enabling the exclusion of xenogeneic Matrigel without negatively affecting the efficiency or proliferation.

### Chemically defined conditions are compatible with DE differentiation in 2D and 3D culture

**Figure 3:**
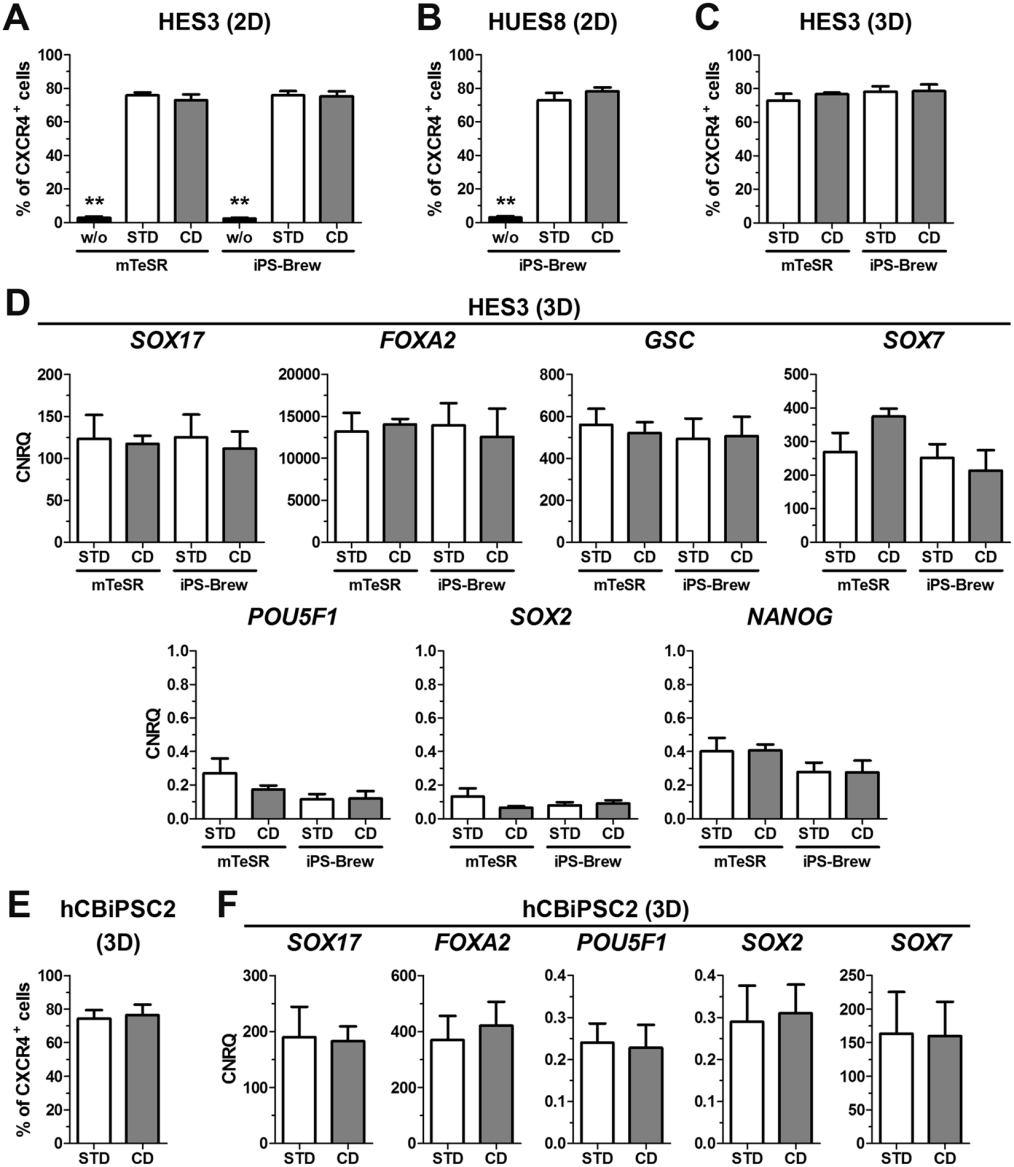
Chemically defined (CD) differentiation in 2D and 3D. HES3, HUES8 or hCBiPSC2 maintained in mTeSR or iPS-Brew were differentiated in 2D or 3D under randomized (w/o), standard (STD) or chemically defined (CD) conditions using 5 µM CHIR. (**A**–**C**) Flow cytometric quantification of CXCR4^+^ cells for HES3 ((**A**), n = 4–8), HUES8 ((**B**), n = 6–8) differentiated in 2D or HES3 differentiated in 3D (**C**, n = 6–8). Values are means ± SEM. Statistical analysis was performed with ANOVA plus *Bonferroni’s* post hoc test, **p < 0.01 compared to all other conditions within the hPSC maintenance media group. (**D**) Normalized expression of *SOX17*, *FOXA2* and *POU5F1* after 3–4 days of 3D differentiation. Values were scaled to undifferentiated cells and represent means ± SEM, n = 6–8. (**E**) Flow cytometric quantification of CXCR4^+^ cells from hCBiPSC2 after four days of 3D differentiation. Values are means ± SEM, n = 4. (**F**) Normalized gene expression of *SOX17*, *FOXA2*, *POU5F1*, *SOX2* and *SOX7* after four days of 3D differentiation scaled to undifferentiated hCBiPSC2 cells. All values are means ± SEM, n = 4. See also Fig. S1.

Differentiation according to the STD protocol was performed in the presence of FCS. In order to establish a chemically defined (CD, Fig. 1) condition, FCS was replaced by our custom made serum-free media supplement called modified (m)B-27 (Table 1)^28^. This mB-27 is related to the commercially available B-27^TM^ but lacks insulin and BSA. The CD condition was tested in 2D (CD-2D) and 3D (CD-3D) culture for its capability to support DE differentiation of hPSCs. The proportion of CXCR4^+^ cells and proliferation under CD-2D and STD-2D conditions were comparable for HES3 and HUES8 cells (Figs 3A,B and S1A,B). As shown for HES3, this was independent of the hPSC maintenance medium (Figs 3A and S1A). Similar, during small scale 3D culture, the CD condition supported DE differentiation of HES3 comparable to the STD condition (Figs 3C and S1C). Gene expression of typical endodermal and pluripotency marker genes during CD-3D and STD-3D conditions were comparable (Fig. 3D). Similar results in 3D culture were also obtained for the human induced pluripotent stem cell (hiPSC) line hCBiPSC2 (Fig. 3E,F). Taken together, replacement of FCS by mB-27 enabled chemically defined differentiation of hPSCs in 2D and 3D culture without negative effects on the DE induction.

**Table 1:** Composition of mB-27 and mB-27XF.

component	concentration in mB-27(XF) [µg/mL]
Biotin	5
L-Carnitine	100
Ethanolamine	50
D-Galactose	750
Putrescine	805
Glutathion (red.)	50
Transferrin	250
Sodium selenite	0,72
Corticosterone	1
Linoleic Acid	50
Linolenic Acid	50
Progesterone	0,32
Retinylacetate	5
Tocopherol	50
Tocopherol acetate	50
Triiodothyronine	0,1
*Catalase*	125
*Superoxide dismutase*	125

mB-27 was prepared as described previously^28^. Bovine catalase and bovine superoxide dismutase (written in italic) that are present in mB-27 were omitted from the formulation of mB-27XF to obtain a xenogeneic-free medium supplement. mB-27 and mB-27XF were applied at a dilution of 1:100 for supplementation of differentiation media.

### Establishment of albumin-free DE differentiation in 2D culture

The CD protocol was based on advanced RPMI 1640 (adRPMI) already supplemented with BSA (AlbuMAX™ II). To establish a BSA-free condition (BF), the adRPMI was replaced by RPMI 1640 (RPMI) or MCDB131 (MCDB) supplemented with BSA-free mB-27.

**Figure 4:**
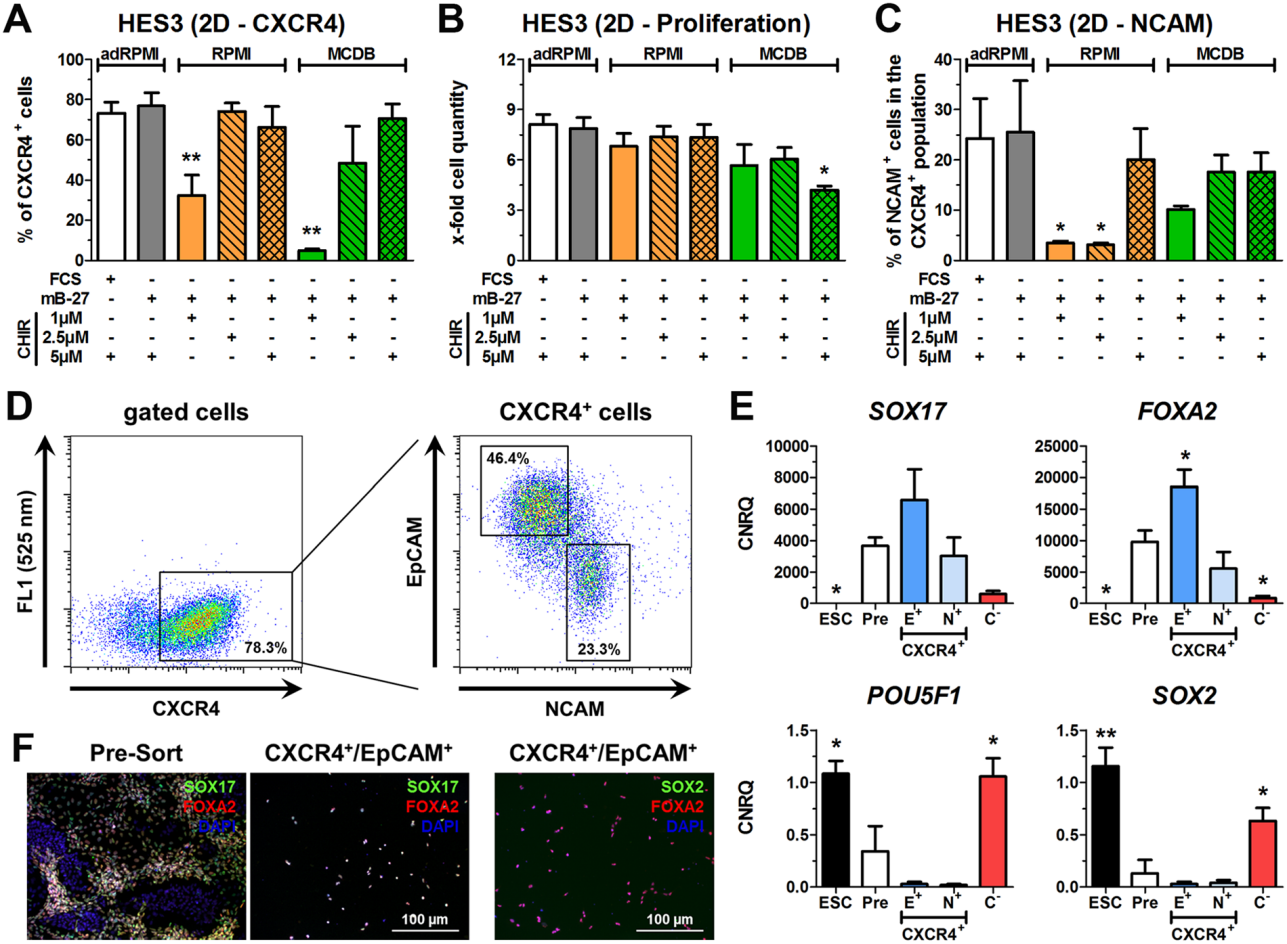
BSA-free (BF) differentiation towards DE in 2D. (**A**–**C**) Differentiation of HES3 in 2D culture in adRPMI, RPMI or MCDB basal medium supplemented with FCS, mB-27 and 1, 2.5 and 5 µM CHIR. Shown are the flow cytometric quantifications of CXCR4^+^ cells (**A**), cell proliferation (**B**) and quantification of NCAM^+^/CXCR4^+^ -positive cells (**C**). All values represent means ± SEM, n = 3–6. Statistical analysis was performed with ANOVA plus *Dunnett’s* post hoc test, *p < 0.05 and **p < 0.01 compared to STD condition (white bar). (**D**) Gating of CXCR4^+^ cells into a CXCR4^+^/NCAM^+^/EpCAM^low^ and a CXCR4^+^/EpCAM^+^ population. (**E**) Normalized expression of *SOX17*, *FOXA2*, *POU5F1* and *SOX2* in undifferentiated HES3 and after four days of differentiation using the STD-2D condition in unsorted cells (Pre) and sorted CXCR4^+^/EpCAM^+^ (E^+^), CXCR4^+^/NCAM^+^/EpCAM^low^ (N^+^) and CXCR4^−^ (C^−^) populations. Values were scaled to undifferentiated cells and represent means ± SEM, n = 3–4. Statistics were performed with ANOVA plus *Dunnett’s* post hoc test, *p < 0.05 and **p < 0.01 compared to unsorted cells (Pre). (**F**) Fluorescence micrographs of SOX17/FOXA2 in pre-sorted cells and SOX17/FOXA2 or SOX2/FOXA2 in CXCR4^+^/EpCAM^+^ sorted cells. Nuclei were counterstained with DAPI (blue). Scale bar: 100 µm. See also Fig. S2.

In line with earlier results^4,5^, the BF-2D condition required a threshold concentration of the Wnt-signaling activator CHIR of at least 2.5 µM during the first 24 h to induce a substantial number of DE cells (Fig. 4A). For all media 5 µM CHIR yielded similar numbers of more than 70% DE committed cells. Interestingly, 2.5 µM CHIR in RPMI (BF-2D) was sufficient to obtain nearly identical numbers of CXCR4^+^ cells compared to the adRPMI-containing controls (STD-2D and CD-2D), while 2.5 µM CHIR in MCDB131 resulted in higher variations (Fig. 4A). Proliferation rates in RPMI (BF-2D) were similar to the adRPMI-containing controls irrespectively of the CHIR concentration, whereas they were significantly reduced with MCDB supplemented with 5 µM CHIR (Fig. 4B).

We also determined the numbers of CXCR4^+^/NCAM^+^ cells (Fig. 4C), which are potentially falsely committed because NCAM is linked to early mesodermal/neuroectodermal differentiation and reorganization of cell assembly^29–32^. Under BSA-free conditions with MCDB medium in 2D, all CHIR concentrations induced a prominent CXCR4^+^/NCAM^+^ population of 10–20% within the CXCR4^+^ cells. BSA-free conditions with RPMI and 1 µM or 2.5 µM CHIR led to significantly lower levels of CXCR4^+^/NCAM^+^ cells compared with adRPMI-containing controls, while 5 µM CHIR considerably increased the percentage of CXCR4^+^/NCAM^+^ cells. Of note, STD-2D and CD-2D conditions with adRPMI and 5 µM CHIR yielded in high levels of CXCR4^+^/NCAM^+^ cells (Fig. 4C). Thus, CHIR appears to induce the appearance of CXCR4^+^/NCAM^+^ cells in a dose-dependent manner.

To characterize this effect, HES3 cells were differentiated using STD-2D conditions with 5 µM CHIR and then stained for CXCR4, NCAM and EpCAM. CXCR4^+^/NCAM^+^/EpCAM^low^ and CXCR4^+^/EpCAM^+^ cells were sorted (Fig. 4D). Remarkably, the CXCR4^+^/EpCAM^+^ population expressed higher levels of *SOX17* and *FOXA2* compared with the pre-sort sample and the CXCR4^+^/NCAM^+^/EpCAM^low^ population (Fig. 4E). Both populations were almost negative for pluripotency genes and expressed the extraembryonic endoderm marker *SOX7* at similar levels (Figs 4E and S2). Interestingly, the CXCR4^+^/NCAM^+^/EpCAM^low^ population exhibited a significant expression of the early primitive streak marker *MIXL1* but a slightly reduced expression of the anterior primitive streak marker *GSC* compared with unsorted cells (pre-sort) and CXCR4^+^/EpCAM^+^ cells (Fig. S2). CXCR4^+^/EpCAM^+^ cells co-expressed SOX17 and FOXA2 on the protein level, while SOX2 was not detectable (Fig. 4F). In contrast, CXCR4^−^ cells expressed similar levels of *POU5F1*, *SOX2* and *NANOG* as undifferentiated hESCs without significant inductions of differentiation markers (Figs 4E and S2). In line with our recent publication^30^, this indicates that the CXCR4^−^ population comprised cells that resisted differentiation.

Taken together, the CXCR4^+^/EpCAM^+^ population represents the favored DE population due to its higher expression of DE marker genes compared with CXCR4^+^/NCAM^+^/EpCAM^low^ cells. Also RPMI as basal medium prevented the formation of NCAM^+^ cells more efficiently than MCDB.

### Establishment of BSA-free DE differentiation in 3D culture

**Figure 5:**
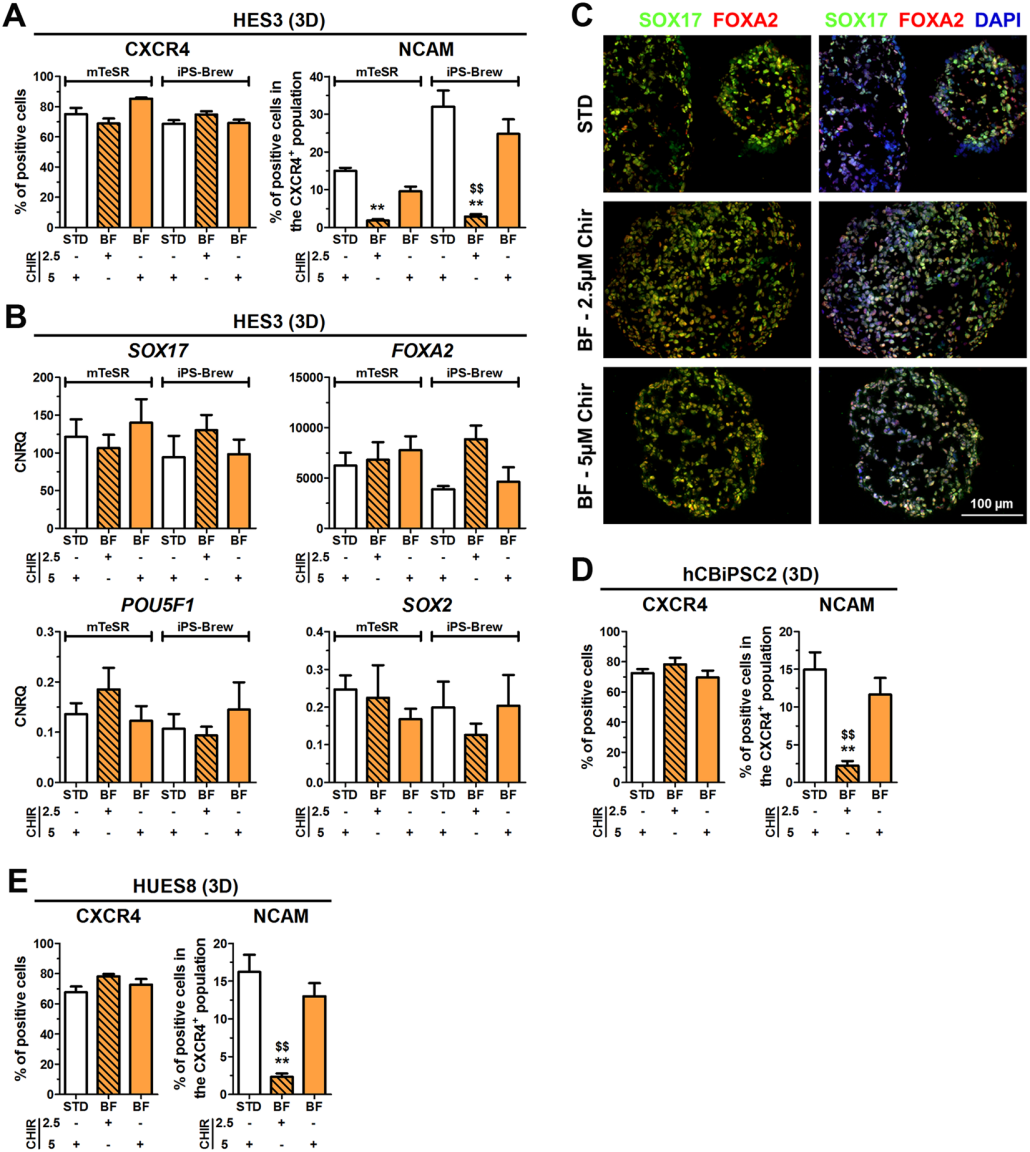
BSA-free (BF) differentiation towards DE in 3D. HES3, HUES8 or hCBiPSC2, maintained in mTeSR or iPS-Brew were differentiated under standard (STD) or BSA-free (BF) conditions using 2.5 or 5 µM CHIR in 3D. (**A**) Quantification of CXCR4^+^ (left) and NCAM^+^/CXCR4^+^ double-positive cells (right) by flow cytometry at day 3/4 upon DE differentiation of HES3. Values are means ± SEM, n = 6–10. Statistical analysis was performed with ANOVA plus *Bonferroni’s* post hoc test, **p < 0.01 compared with STD and ^$$^p < 0.01 comparing 2.5 µM vs 5 µM CHIR using BF conditions. (**B**) Normalized expression of *SOX17*, *FOXA2*, *POU5F1* and *SOX2* upon differentiation of HES3. All values were scaled to undifferentiated cells and represent means ± SEM, n = 6–10. (**C**) Representative fluorescence micrographs of cryosections from cell clusters (HES3) at day 4. Depicted are SOX17 (green) and FOXA2 (red). Nuclei were counterstained with DAPI (blue, right panel). Scale bar: 100 µm. (**D**,**E**) Quantification of CXCR4^+^ cells and of CXCR4^+^/NCAM^+^ cells upon 3D differentiation using hCBiPSC2 (**D**) or HUES8 (**E**) cells. Values are means ± SEM, n = 6–10. **p < 0.01 compared with STD and ^$$^p < 0.01 comparing 2.5 µM vs 5 µM CHIR using BF conditions. See also Fig. S3.

Based on our previous observations (Figs 4 and S2) RPMI was chosen as basal medium to establish a BSA-free 3D culture (BF-3D). Next, the effect of either 2.5 µM or 5 µM CHIR during the first 24 h on the frequency of CXCR4^+^/NCAM^+^ cells was determined. Comparable numbers of CXCR4^+^ cells (~70–80%) were obtained upon 3D differentiation using BF or STD conditions, independent from the hPSC medium (Fig. 5A). As observed in 2D, also under 3D culture, 2.5 µM CHIR resulted in significantly lower CXCR4^+^/NCAM^+^ cells compared to 5 µM CHIR (Fig. 5A). Proliferation rates at BF-3D conditions in the iPS-Brew group were slightly but not significantly lower compared to the STD-3D condition (~4-fold vs. ~4.5-fold, Fig. S3A). However, no significantly different expression levels of *SOX17*, *FOXA2*, *POU5F1*, *SOX2*, *GSC*, *SOX7* and *NANOG* were detectable (Figs 5B and S3B). DE specification was additionally verified by immunofluorescence of SOX17 and FOXA2, which was equally distributed within the cell clusters (Fig. 5C). Similar results were also obtained with the cell lines hCBiPSC2 and HUES8 (Figs 5D,E and S3C,D). In summary, BSA-free 3D differentiation conditions with RPMI as basal medium (BF-3D) induces DE differentiation from hPSCs comparable to the STD-3D condition.

### BSA-free 3D differentiation can give rise to pancreatic progenitor cells

**Figure 6:**
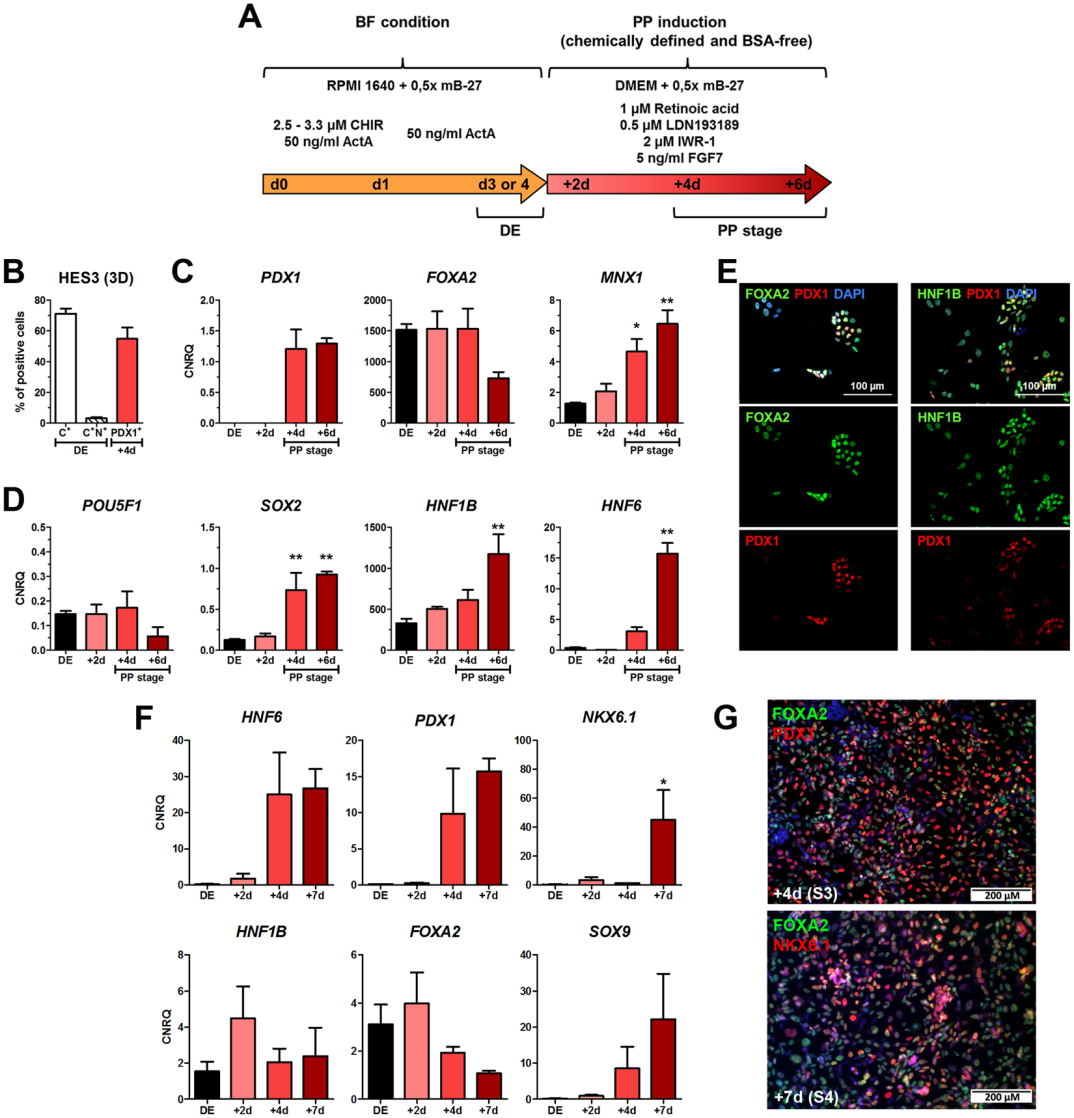
BSA-free (BF) differentiation of HES3 into PDX1^+^ PP cells in 3D. (**A**) Experimental setup for BF-3D differentiation of hPSCs into PP cells. (**B**) Quantification of CXCR4^+^ cells (C^+^) and NCAM^+^ cells within the CXCR4^+^ population (C^+^N^+^) at the DE stage (d3/4) and of PDX1^+^ cells after PP induction (d7/8). Values are means ± SEM, n = 4–6. (**C**) Gene expression of *PDX1*, *FOXA2*, and *MNX1* (*HLXB9*). (**D**) Gene expression of *POU5F1*, *SOX2*, *HNF1B* and *HNF6*. Values in (**C**) and (**D**) are means ± SEM, n = 3–4. (**E**) Representative fluorescence micrographs at day 7/8 for PDX1/FOXA2 and PDX1/HNF1B after dissociation and re-seeding on chamber slides one day prior fixation. Nuclei were stained in blue. Scale bar: 100 µM. (**F**) Gene expression changes of *HNF6*, *SOX9*, *PDX1*, *HNF1B*, *FOXA2* and *NKX6*.*1* upon differentiation of DE clusters obtained by XF-3D according to the Kieffer-protocol^30^. Values are means ± SEM n = 3. (**G**) Representative fluorescence micrographs of FOXA2/PDX1 at the end of stage 3 (+4d) and FOXA2/NKX6.1 at the end of stage 4 (+7d). Nuclei were stained in blue. Scale bar: 200 µM. Statistics were performed with ANOVA plus *Dunnett’s* post hoc test, **p < 0.01, *p < 0.05 compared to DE. See also Fig. S4.

Next, we determined whether BF-2D/3D culture permits differentiation into PDX1^+^ pancreatic progenitor (PP) cells using our previously published protocol^27^ adapted to BSA-free differentiation (Fig. 6A). HCBiPSC2 cells were differentiated under BF-2D into DE resulting in >80% CXCR4^+^ cells. Subsequent differentiation for four days yielded ~60% PDX1^+^ PP cells (Fig. S4A). *PDX1* expression became strongly detectable and *FOXA2* remained strongly expressed throughout the protocol (Fig. S4B). Contemporaneously, expression of *POU5F1* decreased, while expression of *SOX2* continuously increased during differentiation of DE into PP cells (Fig. S4B).

Next, these conditions were tested in 3D with a different cell line (HES3). From ~70% CXCR4^+^ cells >50% PDX1^+^ PP cells were generated after four days of PP induction (Fig. 6B). *PDX1*, *FOXA2*, *MNX1*, *SOX2*, *HNF1B*, and *HNF6* gene expression was strongly increased or maintained during PP induction (Fig. 6C,D). Similar to 2D culture conditions, *POU5F1* expression was very low, (Fig. 6D). Furthermore, co-expression of PDX1 and FOXA2 demonstrated the endodermal origin of the PDX1^+^ cells, whereas the co-expression of PDX1 with HNF1B revealed foregut identity (Fig. 6E). Finally we tested differentiation of DE cells generated at XF-3D conditions into multipotent pancreatic progenitor cells positive for NKX6.1. Therefore, the resulting clusters were allowed to settle down and treated for the next seven days of differentiation according to the Kieffer-protocol^33^. Typical pancreatic marker genes including *NKX6*.*1* were strongly expressed upon differentiation (Fig. 6F). Furthermore PDX1- (S3) and NKX6.1-positive cells (S4) were readily detected by immunofluorescence (Fig. 6G). Thus, 3D BSA-free generated DE is capable of pancreatic differentiation.

### hPSCs can be differentiated into DE under xenogeneic-free conditions

**Figure 7:**
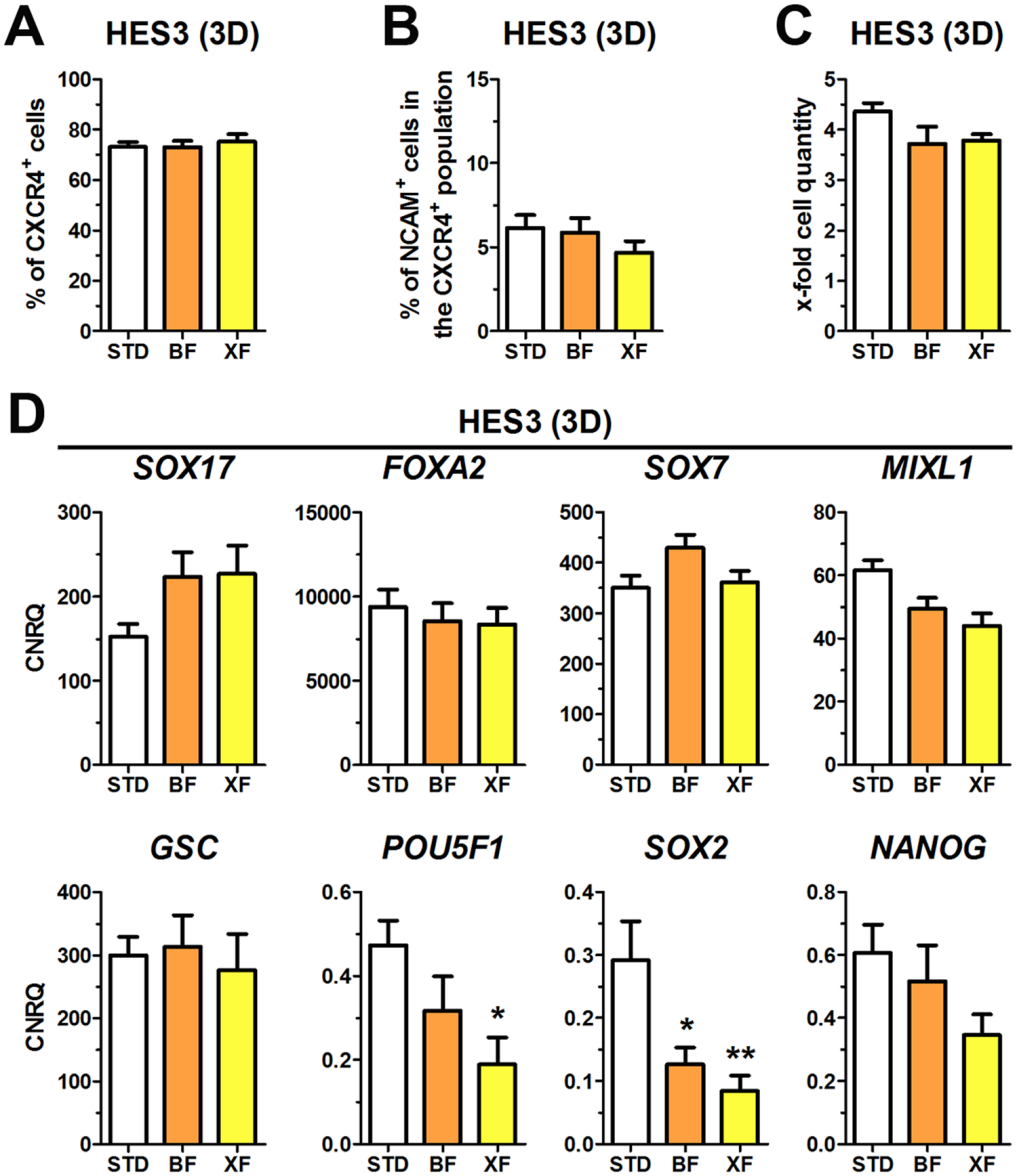
Xenogeneic-free (XF) differentiation in 3D towards DE. (**A**–**D**) Differentiation of HES3 in 3D culture under standard (STD), BSA-free (BF) or xenogeneic-free (XF) conditions towards DE showing the flow cytometric quantification of CXCR4^+^ cells (**A**), quantification of NCAM/CXCR4 double-positive cells (**B**) and cell proliferation (**C**). All values represent means ± SEM, n = 4–8. (**D**) Normalized expression of the marker genes *SOX17*, *FOXA2, SOX7*, *MIXL1*, *GSC*, *POU5F1*, *SOX2* and *NANOG* after four days of differentiation at the indicated conditions. All values are scaled to undifferentiated cells and represent means ± SEM, n = 4–8. Statistic was performed with ANOVA plus *Dunnett’s* post hoc *test*, *p < 0.05 and **p < 0.01 compared to the STD condition (white bar). See also Fig. S5.

Our custom-made mB-27 still contained bovine superoxide dismutase and catalase. In order to exclude any xenogeneic compound from the medium, these two enzymes were removed from mB-27 resulting in a xenogeneic-free formulation designated as mB-27XF (Table 1). In addition, activin A was dissolved in pure water without BSA for protein stabilization. This resulted in a 100% chemically defined and xenogeneic-free media formulation for hPSCs differentiation into DE designated XF. XF was compared to STD and BF for HES3 and HUES8 under 2D culture (XF-2D, Fig. S5) and for HES3 under 3D culture (XF-3D, Fig. 7). Under 2D and 3D culture the percentages of CXCR4^+^ cells and the proportion of unwanted CXCR4^+^/NCAM^+^ cells were similar for all tested conditions (Figs 7A,B and S5A,B). We also tested the effects of 1, 2.5, and 5 µM CHIR on the distribution of EpCAM^+^, NCAM^+^ and CXCR4^+^ cells under XF conditions during differentiation and verified CXCR4 gene and protein expression by RT-qPCR and Western Blot (Fig. S6A–C). As described above, higher CHIR concentrations (>2.5 µM) yielded in a NCAM^+^ cell population slightly expressing EpCAM (EpCAM^low^) at the expense of EpCAM^+^ cells. Analysis of CXCR4 expression in EpCAM^+^ and NCAM^+^/EpCAM^low^ cells revealed that CXCR4 is upregulated in both populations dependent on the CHIR concentration. In NCAM^+^/EpCAM^low^ cells, however, CXCR4 is weakly expressed along with lower gene expression of *FOXA2* and *SOX17* (Fig. 4). Thus, these NCAM^+^/EpCAM^low^/CXCR4^low^ cells likely represent misspecified endoderm cells as a result of Wnt/beta-catenin hyperactivation (Fig. S6A). Further representative flow cytometry dot plots are presented in Supplementary Figs S7 and S8.

The proliferation rates in the 3D culture were unaltered comparing the tested conditions (Fig. 7C). The XF condition did not influence expression levels of *SOX17*, *FOXA2*, *SOX7*, *MIXL1* and *GSC* under 3D or 2D culture compared to the BF and STD conditions. However, expression of pluripotency markers (*POU5F1*, *SOX2*, *NANOG*) appeared to be slightly reduced after four days of DE differentiation with the XF condition (Figs 7D and S5C,D).

## Discussion

Differentiation media for hPSCs are typically not defined and contain xenogeneic components that harbor risks of transmitting pathogenic agents such as zoonotic viruses or mycoplasma to human recipients^34,35^. Additionally, the incorporation of foreign proteins or glycans^36,37^ into hPSC-derived lineages may possibly activate the recipient’s immune system and cause graft inflammation or rejection. Removal of xenogeneic compounds from differentiation media would also simplify the governmental registration of stem cell-derived medical products and disburden animals from pain as requested by the 3 R principle^38^. Furthermore, chemically defined media compositions, in which all ingredients are precisely known, allow standardization with reduced variabilities during cultivation and differentiation. Consequently, the development of chemically defined and xenogeneic-free differentiation protocols for hPSCs is an important step for future clinical application of hPSC-derived somatic cells. To achieve this aim, chemically undefined and xenogeneic components including Matrigel, FCS and BSA were removed step-by-step from our previously published DE and PDX1^+^ PP differentiation protocols^4,27^.

In order to eliminate Matrigel, hPSCs were cultured in 3D as free floating spheroids in the absence of exogenous extracellular matrices or scaffolds as described before^39^ and were then differentiated into DE cells with similar efficiencies as reported for standard 2D culture conditions^4^. In line with our observations, efficient DE induction in 3D suspension culture of hPSC spheroids has been already described by Pagliuca *et al*.^24^, which demonstrates that self-assembled suspension culture spheroids of hPSCs provide an efficient 3D environment without the need for exogenous matrices. The possibility to scale up 3D cultures to controlled, stirred bioreactors^25,26^ enables the mass expansion and differentiation of hPSCs under defined conditions to obtain abundant cell quantities needed for (pre)clinical applications. Our standard medium for DE differentiation^4^ contained FCS and BSA. Both are xenogeneic components and can be found in various DE differentiation media^8,14,40^. Also the recently published 3D differentiation conditions by Pagliuca *et al*. contained BSA^24^. It has been earlier shown that replacement of serum by the chemically defined and serum-free supplement B-27^TM^ was compatible with efficient DE differentiation of hPSCs^9,10,16^. To obtain chemically defined conditions for 2D differentiation into DE, Teo *et al*. further replaced the Matrigel by fibronectin^9,10^. However, commercial B-27^TM^ present in these differentiation media contains high concentrations of BSA that is commonly derived by fractionation of serum proteins and it is neither pure nor defined. With the aim to omit BSA and because the commercial B-27^TM^ components’ concentrations are still confidential, we generated a custom-made modified B-27 (mB-27, Table 1)^28^. This mB-27 is based, with minor modifications, on the formulation of B-27 and its ancestor B-18^21,41,42^ but lacks BSA and insulin. A further source of BSA in our standard DE differentiation medium (STD) was the adRPMI, which was in a subsequent step replaced by standard, BSA-free RPMI. Both, replacement of B-27^TM^ and adRPMI by mB-27 and RPMI, respectively, in combination with a Matrigel-free 3D culture condition yielded in a chemically defined (CD) and BSA-free (BF) differentiation medium that supported DE differentiation comparable to the standard condition. These findings appear contrary to Wang *et al*., who could substitute B-27^TM^ with BSA although they found that BSA alone “was not as good as complete B-27”^16^. Different to Wang *et al*., our BSA-free mB-27 contained the other ingredients of B-27, including vitamins, antioxidants, lipids, steroids and trace elements. ITS-X (insulin, transferrin, selenite and ethanolamine) was separately supplemented to our differentiation media, which might compensate for the loss of BSA and insulin. Especially insulin is generally considered as a potent mitogen^43,44^ that might in our approach support cell survival. Accordingly, BSA-free differentiation of hPSCs towards DE has been recently described by Qu and co-workers^17^ in the absence of cell viability supporting supplements like serum, BSA, B-27^TM^ or insulin by using a cocktail of activin A, WNT3A, BMP4 and bFGF that prevented activin A induced cell death. However, in their chemically defined (CD) 2D culture system it was not clear whether fibronectin/vitronectin was of xenogeneic origin.

Interestingly, we observed in BF and XF conditions less NCAM^+^ cells within the CXCR4^+^ population in RPMI medium at lower CHIR concentrations. Typical DE genes were significantly reduced in CXCR4^+^/NCAM^+^/EpCAM^low^ compared with CXCR4^+^/EpCAM^+^ cells. Thus, these cells might represent a falsely specified endoderm subpopulation. CXCR4^+^/NCAM^+^/EpCAM^low^ cells strongly expressed *MIXL1*, whereas *GSC* was reduced. This indicates that this population could be stuck in a primitive streak pattern or was partially committed towards a mesodermal cell fate. Accordingly, Kempf *et al*. recently proposed a model, in which the concentration of CHIR and its duration affects primitive streak-like patterning along the anterior-posterior axis influencing cell fate decisions towards DE, cardiac mesoderm or presomitic mesoderm^45^. Along with Lian *et al*., who reported that BSA reduces the activity of CHIR during mesodermal differentiation^46^ and the issue that NCAM-positivity in the CXCR4^+^ population increased in a CHIR concentration-dependent manner, the CHIR concentration should be carefully determined for BSA-free differentiation conditions. Furthermore, our data suggests NCAM-positivity as a negative marker for DE quality.

DE cells obtained by the newly established BF condition for DE formation in 2D and 3D culture could be further differentiated into PDX1^+^ pancreatic progenitor (PP) under BSA-free conditions adapted from our recently published protocol^27^ and into multipotent pancreatic NKX6.1^+^ progenitor cells according to the Kieffer-protocol^33^. Actually, 3D culture was shown to be superior to 2D culture for further maturation of DE cells into PDX1^+^ PP cells and subsequently into insulin producing cells^24,47,48^, which underlines the importance of scalable 3D cultures. It can be expected that the microenvironment within these 3D aggregates mimics the *in vivo* distribution and accessibility of morphogens and nutrients and thus may enhance maturation of differentiated progeny^49,50^. In line with our findings, Pagliuca *et al*. reported the efficient differentiation of 3D suspension culture spheroids of hPSCs into PDX1^+^ PP cells and into functional pancreatic beta cells^24^. In the future fully defined and xenogeneic-free differentiation protocols for the generation of pancreatic beta cells will be required.

As our mB-27 still contained a small amount of bovine-derived proteins (superoxide dismutase and catalase), we also omitted these proteins to obtain a 100% xenogeneic-free formulation designated mB-27XF. This mB-27XF supported DE differentiation to a similar extent as the CD, BF or STD condition, which is in concordance with Wang *et al*., who reported that antioxidants in B-27^TM^ were redundant during DE differentiation of hPSCs^16^.

In summary, we describe here the first fully chemically defined and xenogeneic-free differentiation protocol in 3D for various hPSC lines permitting efficient DE differentiation.

## Methods

### Human PSC culture

HESC lines HES3 and HUES8 and the hiPSC line hCBiPSC2^51^ were cultivated as described earlier^4,27,30^. Briefly, all hPSCs were cultured as colonies on cell culture plastic coated with hESC-qualified Matrigel (Corning, Amsterdam, Netherlands). As cultivation medium mTeSR1 (Stem Cell Technologies, Cologne, Germany) or StemMACS™ iPS-Brew XF (Miltenyi Biotec, Bergisch Gladbach, Germany) was used. Passaging was performed every 5–7 days using a non-enzymatic solution^52^ or Dispase (5 U/mL) in a ratio of 1:6 up to 1:40 onto Matrigel-coated 6-well plates.

### Formulation of modified B-27 (mB-27) and xenogeneic-free modified B-27 (mB-27XF)

Our custom made modified B-27 (mB-27)^28^ is based on a publication by Roth *et al*.^42^. with modifications. Substances are specified in Table 1. 50x stock solutions of modified B-27 (mB-27) or the xenogeneic-free modified B-27 (mB-27XF) were assembled, sterile-filtered and stored at −20 °C in aliquots. After thawing mB-27 or mB-27XF aliquots were diluted to a final concentration of 0.5x in the culture media.

### Differentiation experiments

Differentiation of hPSCs as adherent monolayer (2D) was performed as described earlier^4^ and the media composition will be herein referred to as standard (STD) condition. Briefly, hPSC colonies were dissociated into single cells by Trypsin/EDTA (Biochrom) and centrifuged for 3 min at 300 × g. The pellet was re-suspended in mTeSR1 or iPS-Brew XF containing 10 µM Y-27632 (Selleck Chemicals, Munich, Germany) and a defined number (50,000–70,000 cells/cm^2^ for HES3 and hCBiPSC2; 70,000–90,000 cells/cm^2^ for HUES8) was seeded on cell culture dishes coated with used or fresh Matrigel. Cells were allowed to re-attach overnight and differentiation was initiated the following day.

For DE differentiation in static suspension (3D) culture, HES3 or hCBiPSC2 were dissociated into single cells either using 0.5 µM EDTA (in PBS) or gentle cell dissociation reagent (Stem Cell Technologies). A defined cell number was inoculated in Costar^®^ Ultra-Low Attachment 6-well plates (Corning) using mTeSR1 or iPS-Brew XF supplemented with 10 µM Y-27632. Initially tested cell seeding densities ranged from 0.25 to 1 × 10^6^ cells per well. For subsequent experiments 1–2 × 10^6^ cells per well were used as inoculum. HUES8 cells were seeded as small clusters in a similar size as obtained during normal passaging. One full well of a 6-well plate (~2–3 million cells) was re-seeded into 2–3 wells of a Costar^®^ Ultra-Low Attachment 6-well plate. All hPSC lines were cultured o/n or for 24–48 h in maintenance medium to permit aggregate formation before differentiation.

DE differentiation was performed by medium supplemented with 1, 2.5 and 5 µM CHIR-99021 (CHIR) (Biozol, Eching, Germany) and 50 ng/ml activin A (Peprotech, Hamburg, Germany) for 24 h followed by 48–72 h with 50 ng/ml activin A only. Media were changed daily. Randomized differentiation was performed without adding CHIR and activin A. DE differentiation of hPSCs was performed in different basal media. All basal media were supplemented with 1x GlutaMAX^TM^ or 2 mM glutamine plus penicillin/streptomycin (P/S, 10 IU/ml and 70 IU/ml). Instead of advanced RPMI 1640 (adRPMI, Thermo Fisher Scientific) RPMI 1640 (RPMI, Biochrom, Berlin, Germany) was used and supplemented with 0.11 mg/ml sodium pyruvate (Sigma Aldrich, Munich, Germany), 1x ITS-X (Thermo Fisher Scientific) and 1x non-essential amino acids (NEAA, Thermo Fisher Scientific), while MCDB131 additionally contained 1x NEAA, 1x ITS-X and 5.5 mM glucose. Basal media were further supplemented with 0.2% FCS (PAA, Vienna, Austria), mB-27 or mB-27XF and designated according to Fig. 1.

After DE induction pancreatic differentiation for 6 days was performed with minor modifications as earlier described^27^. The basal medium was changed to DMEM (Biochrom) supplemented with P/S, glutamine, 0.5x mB-27, 1 µM all-trans retinoic acid (Sigma-Aldrich), 0.5 µM LDN-193189 (Sigma-Aldrich), 2 µM IWR-1 (Selleck Chemicals) and 5 ng/ml FGF-7 (ReliaTech, Wolfenbüttel, Germany). Differentiation into NKX6.1-expressing cells was done according to Rezania *et al*.^33^. Clusters generated in BF- 3D from HES3 cells were allowed to settle down from day 3–4 and then differentiation was continued in S2 (+2d), S3 (+4d) and S4 (+7d) medium with minor modifications (Pdbu was used instead of TPB).

### Flow cytometry and cell sorting

Cells in 2D conditions were washed with PBS and dissociated using Trypsin/EDTA or 0.5 µM EDTA. Clusters from 3D suspension culture were collected in a 15 ml conical tube, centrifuged at 300 × g for 3 min and subsequently dissociated by incubation with 0.5–1 ml collagenase B and occasional tapping (1 mg/mL, diluted in low calcium buffer, Table S1) for 7–10 min. Single cells were then centrifuged at 300x g for 3 min and re-suspended in PBS + 2% FCS.

For flow cytometric staining 1–2 × 10^5^ cells were washed, incubated for 20–60 min at 4 °C with primary conjugated antibodies and washed twice prior to analysis. Staining of intracellular PDX1 was performed with the Foxp3 staining buffer set (Thermo Fisher Scientific). Flow cytometric measurement was performed on a CyFlow ML flow cytometer (Partec, Münster, Germany) and at least 2 × 10^4^ events of each sample were analyzed using the FlowJo software (Ashland, OR, USA). Fluorescence activated cell sorting (FACS) was performed at the central facility of the Hannover Medical School.

The following anti-human Fc-conjugated antibodies were used: CXCR4-PE (FC15004, Neuromics, Minneapolis, USA), CXCR4-APC (130-098-357, Miltenyi Biotec), CD56/NCAM-BV510 (318340, BD Biosciences, Heidelberg, Germany), NCAM-FITC (345811, BD Biosciences), CD326/EpCAM-PE (135905, BioLegend, San Diego, CA, USA) and CD56/NCAM-BV510 (318340, BD Bioscience, Heidelberg, Germany). For staining of PDX1 the primary PDX1 antibody (AF2419, R&D Systems, Minneapolis, USA) was visualized with a secondary AlexaFluor-647-conjugated antibody (Dianova, Hamburg Germany).

### Gene expression analysis

Isolation of total RNA was carried out using the peqGOLD Total RNA Kit (VWR International, Erlangen, Germany). CDNA was synthesized from 500–2000 ng total RNA using RevertAid™ H Minus M-MuLV Reverse Transcriptase (Thermo Fisher Scientific) and random hexamer primers. CDNA samples were then diluted to 5–10 ng/µl and measured in a PCR reaction with the GoTaq^®^ qPCR Master Mix (Promega). All reactions were performed by a 2-step PCR in triplicates followed by melting curve analysis on a ViiA7 real-time PCR cycler (Thermo Fisher Scientific). Primer pairs are specified in Table S2. Data normalization was performed with qBasePlus (Biogazelle, Zwijnaarde, Belgium) against the geometric mean of the three housekeeping genes *G6PD*, *TBP* and *TUBA1A*. Analysis of housekeeping gene stability was performed with the geNorm algorithm.

### Immunocytochemistry

Sorted or dispersed cells were centrifuged for 3 min at 300x g, re-suspended in differentiation medium supplemented with 10 µM Y-27632 and seeded onto Matrigel-coated glass cover slides (SPL Life Sciences, Pocheon, South Korea). After 24 h the cells were fixated for 10–30 min with 4% (w/v) paraformaldehyde.

Clusters obtained by 3D differentiation were collected with a wide-bore tip and washed once with PBS. Fixation was performed as described above. Embedding of the cluster was done in cryomolds (Sakura, Alphen an den Rijn, Netherlands) with Tissue-Tek^®^ O.C.T.™ Compound (Sakura). Subsequently, 5 µm cryosections were cut with a cryostat CM3050 (Leica Biosystems, Nussloch, Germany) and transferred onto superfrost plus slides at −22 °C (Thermo Fisher Scientific).

For immunocytochemistry, cryosections and glass cover slides were washed once with PBS and then blocking was performed for 20 min in PBS plus 0.2% Triton X-100 and 1 mg/ml NaBH_4_ with 6% BSA or 5% donkey serum (Dianova). Primary and secondary antibodies were diluted in PBS with 0.1% Triton X-100 plus 0.1% BSA. Primary antibodies were incubated for 1–3 h at room temperature or overnight at 4 °C. Secondary antibodies were diluted 1:250–1:500 and incubated for 1 h at room temperature. The following antibodies were used: anti-SOX17 (AF1924, R&D Systems, Minneapolis, USA), anti-FOXA2 (07–633, MerckMillipore, Schwalbach, Germany), anti-SOX2 (sc-17320, SantaCruz, Dallas, USA), anti-PDX1 (AF2419, R&D Systems), anti NKX6.1 (AF5857, R&D Systems), and anti-HNF1b (sc-22840, Santa Cruz). Secondary antibodies were obtained from Dianova conjugated with AlexaFluor or Cy fluorophores. Finally, the slides were mounted with immunoselect anti-fading mounting medium (Dianova) containing DAPI. Stained cells were examined using an Olympus IX81 microscope (Olympus, Hamburg, Germany) and multiple representative pictures were taken of each sample.

### Statistics

Unless stated otherwise values represent mean ± SEM and the number of independent experiments (n) is stated in the figure legend. Statistical analyses were performed using the GraphPad Prism analysis software (Graphpad, San Diego, CA, USA) using *ANOVA* followed by *Bonferroni’s* or *Dunnett’s* post-hoc-test for multiple comparisons.

## Supplementary information

Supplementary data
