# Supplementary material for: Chemically defined and xenogeneic-free differentiation of human pluripotent stem cells into definitive endoderm in 3D culture
Source: Sci Rep. 2019 Jan 30;9:996. doi: 10.1038/s41598-018-37650-z (PMC6353891; doi:10.1038/s41598-018-37650-z)
Supplement: Supplementary file 1 — Supplementary data [file 41598_2018_37650_MOESM1_ESM.docx]

**Supplementary Information**

**Chemically defined and xenogeneic-free differentiation of human pluripotent stem cells into definitive endoderm in 3D culture**

Ulf Diekmann^1^, Hanna Wolling^1,2^, Rabea Dettmer^1^, Isabell Niwolik^1^, Ortwin Naujok^1,*,#^ & Falk F. R. Buettner^1,2,*,#^

^1^ Institute of Clinical Biochemistry, Hannover Medical School, Hannover, Germany

^2^ REBIRTH Cluster of Excellence, Hannover Medical School, Hannover, Germany

***Corresponding and # equally contributing authors**

***Prof. Dr. Falk F. R. Buettner***

Institute of Clinical Biochemistry

Hannover Medical School

30625 Hannover

Germany

Phone: +49/511/532-8245

Fax: +49/511/532-8801

E-mail: buettner.falk@mh-hannover.de

***PD Dr. Ortwin Naujok***

Institute of Clinical Biochemistry

Hannover Medical School

30625 Hannover

Germany

Phone: +49/511/532-3544

Fax: +49/511/532-3584

E-mail: naujok.ortwin@mh-hannover.de


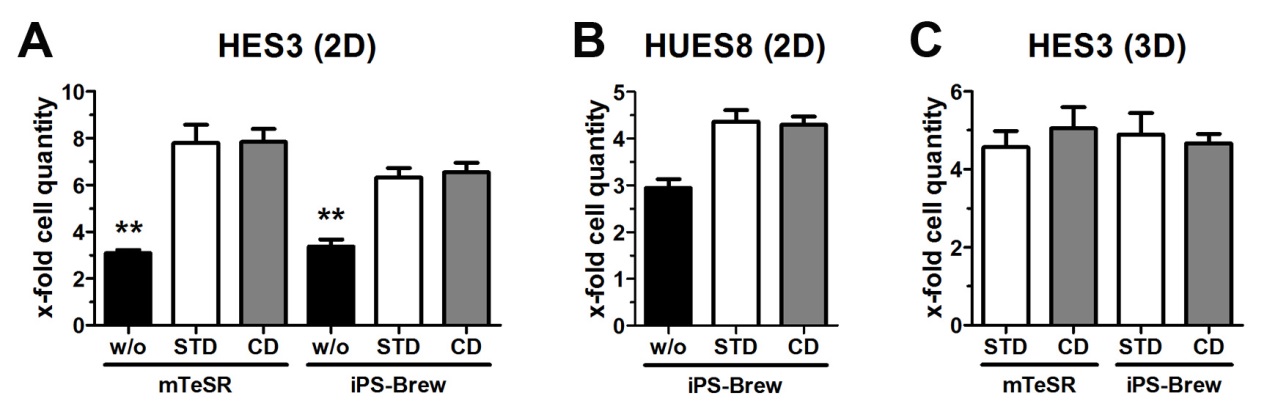


**Figure S1 │ Cell proliferation upon chemically defined (CD) differentiation in 2D and 3D.** (**A-C**) Increase of cell numbers upon randomized (w/o) or directed DE differentiation according to the CA-A approach (STD) or chemically defined (CD) conditions in 2D or 3D for different cells lines (HES3, HUES8) that were maintained as indicated in mTeSR or iPS-Brew. Statistical analysis was performed with ANOVA plus *Bonferroni’s* post hoc test (**A**, **B**) or with *Students t-test* (**C**), **p < 0.01 compared with all other conditions within the maintenance media group.


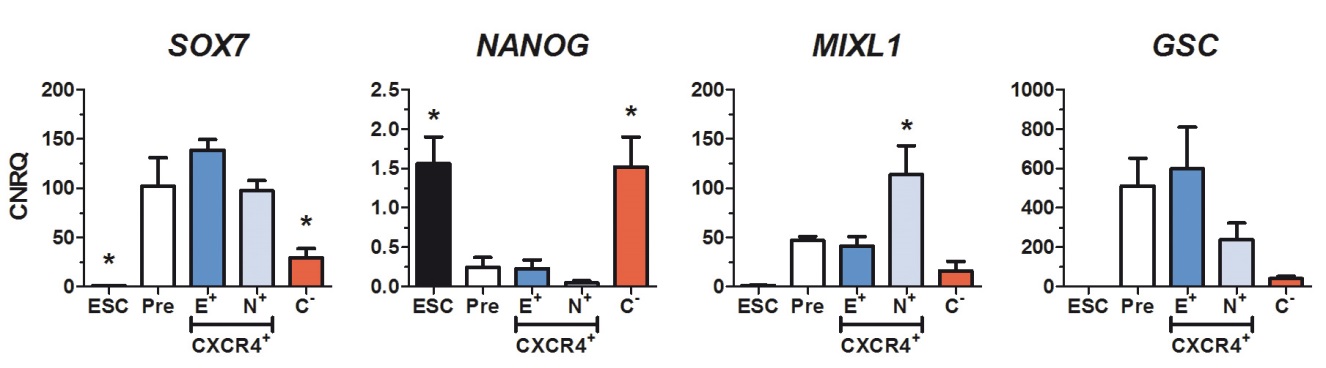


**Figure S2 │ Expression of marker genes dependent on CXCR4 and NCAM expression.** Normalized expression of *SOX7*, *NANOG*, *MIXL1* and *GSC* in undifferentiated HES3 (ESC) and after four days of differentiation at STD-2D condition in unsorted cells (Pre) and sorted CXCR4^+^/EpCAM^+^ (E^+^), CXCR4^+^/NCAM^+^ (N^+^) and CXCR4^-^ (C^-^) populations. Values represent means ± SEM, n = 3-4. Statistical anaylsis was performed with ANOVA plus *Dunnett’s* post hoc test, *p < 0.05 and **p < 0.01 compared with the unsorted sample (Pre).


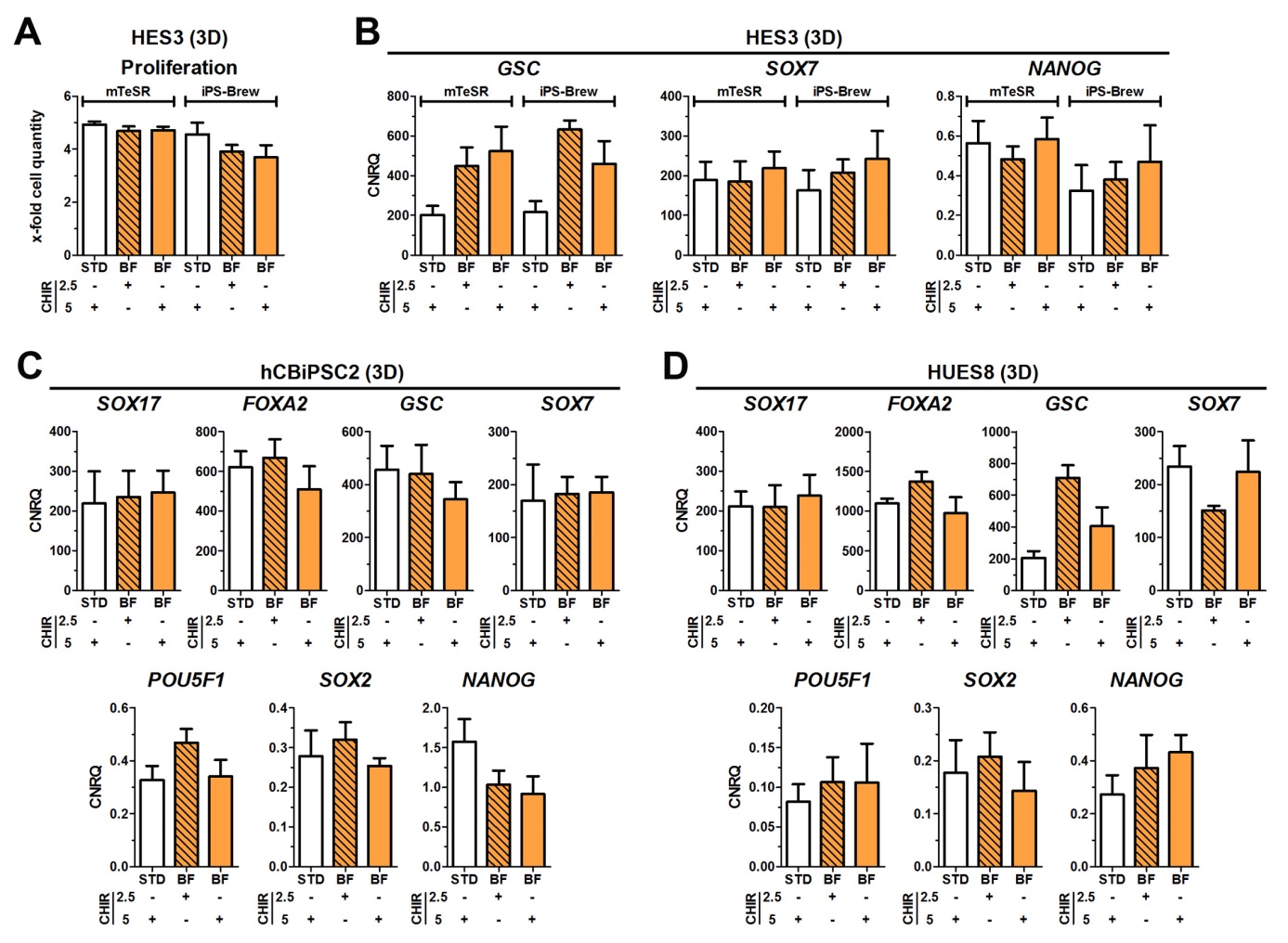


**Figure S3 │ BSA-free (BF) differentiation towards DE in 3D.** HES3, HUES8 and hCBiPSC2 maintained in mTeSR or iPS-Brew were differentiated under standard (STD) or BSA-free (BF) conditions using 2.5 or 5 µM CHIR in 3D. (**A**) Cell proliferation of HES3 cells in 3D culture. (**B**) Normalized expression of *GSC*, *SOX7* and *NANOG* upon differentiation of HES3 in 3D culture. All values were scaled to undifferentiated cells and represent means ± SEM, n = 6-10. (**C-D**) Normalized expression of endodermal (*SOX17*, *FOXA2*), extra-embryonic endoderm (*GSC*, *SOX7*) and pluripotency (*POU5F1*, *SOX2, NANOG*) marker genes upon differentiation of hCBiPSC2 (**C**) and HUES8 (**D**) in 3D culture. All values were scaled to undifferentiated cells and represent means ± SEM, n = 6-10.


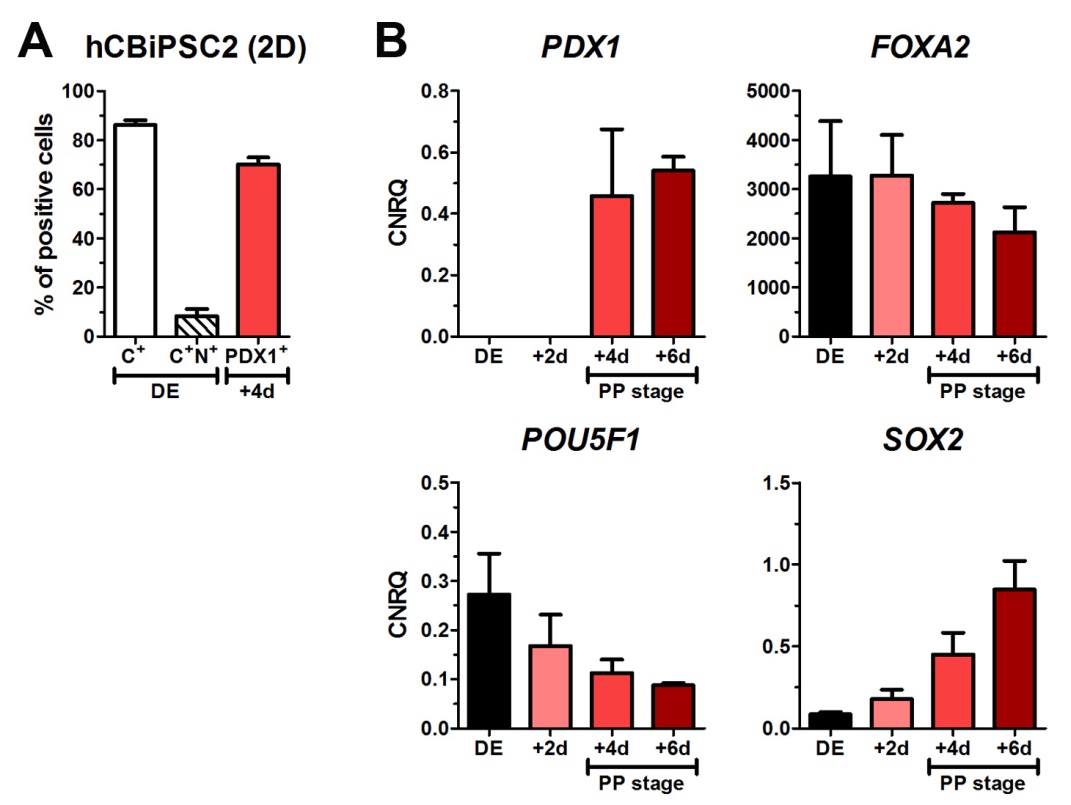


**Figure S4 │ BSA-free (BF) differentiation of hCBiPSC2 into PDX1+ PP cells.** (**A**) Quantification of CXCR4^+^ cells (C^+^) and NCAM^+^ cells within the CXCR4^+^ population (C^+^N^+^) at the DE stage (d3 or d4) and of PDX1^+^ cells after PP induction for 4 days (d7 or d8). Values are means ± SEM, n = 4. (**B**) Normalized gene expression of the pan-pancreatic marker *PDX1*, the endodermal marker *FOXA2* and pluripotency markers (*POU5F1*, *SOX2*). Values are means ± SEM, n = 4.


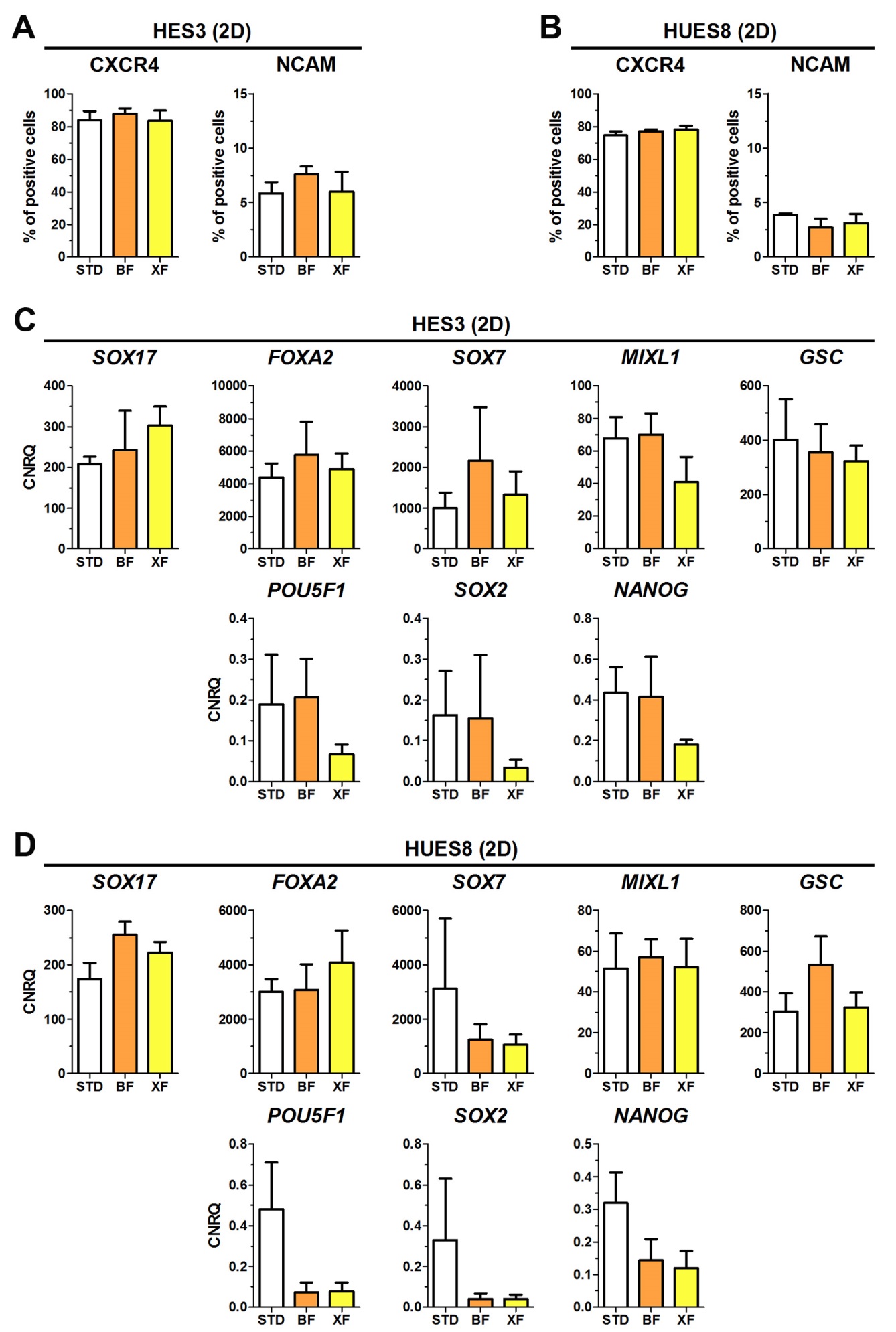


**Figure S5 │ Xenogeneic-free (XF) differentiation in 2D towards DE.** (**A-D**) Differentiation of HES3 or HUES8 in 2D culture under standard (STD), BSA-free (BF) or xenogeneic-free (XF) conditions towards DE. (**A-B**) Flow cytometric quantification of CXCR4^+^ and CXCR4^+^/NCAM^+^ cells upon differentiation of HES3 (**A**) or HUES8 (**B**) in 2D culture using the indicated conditions. All values represent means ± SEM, n = 3. (**C-D**) Normalized gene expression of *SOX17*, *FOXA2*, *SOX7*, *MIXL1*, GSC, POU5F1, *SOX2* and *NANOG* after four days of differentiation using HES3 (**C**) or HUES8 (**D**) cells. All values represent means ± SEM, n = 3 and were scaled to undifferentiated cells. Statistical analysis was performed with ANOVA plus *Dunnett’s* post hoc test, *p < 0.05 and **p < 0.01 compared to the STD condition (white bar).


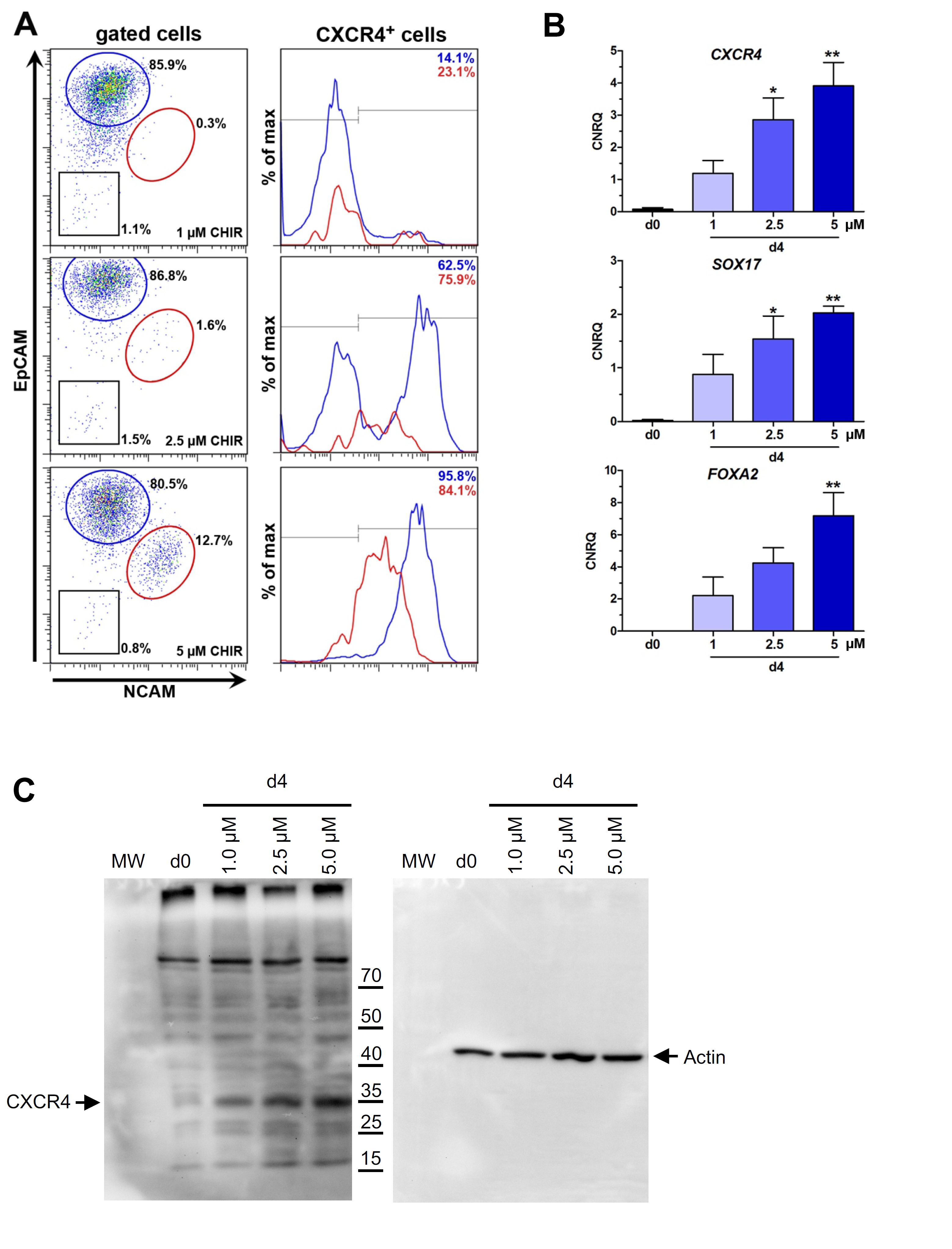


**Figure S6 │ Xenogeneic-free (XF) differentiation in 3D towards DE.** Differentiation of HES3 in 3D culture under XF conditions towards DE at 1, 2.5 and 5 µM CHIR (**A-C**) and changes of EpCAM, NCAM and CXCR4 expression measured by flow cytometry (**A**). Differentiated cells at d4 were stained with EpCAM-PE, NCAM-FITC and CXCR4-APC. Higher CHIR concentrations (> 2.5 µM) yielded in a NCAM^+^ cell population slightly expressing EpCAM (red gate, EpCAM^low^) at the expense of EpCAM^+^ cells (blue gate). CXCR4 is upregulated in both populations dependent on the CHIR concentration (red and blue histogram). In NCAM^+^/ EpCAM^low^ cells CXCR4 is weaker expressed (**A**). (**B-C**) Gene (*CXCR4*, *SOX17* and *FOXA2*) and protein expression (CXCR4) upon differentiation in comparison to undifferentiated cells (d0) measured by RT-qPCR (**B**) and Western blot (**C**) in presence of 1, 2.5 and 5 µM CHIR. RT-qPCR values represent means ± SEM, n = 6 and were scaled to average. Statistic was performed with ANOVA plus *Dunnett’s* post hoc test, *p < 0.05 and **p < 0.01 compared to the d0.


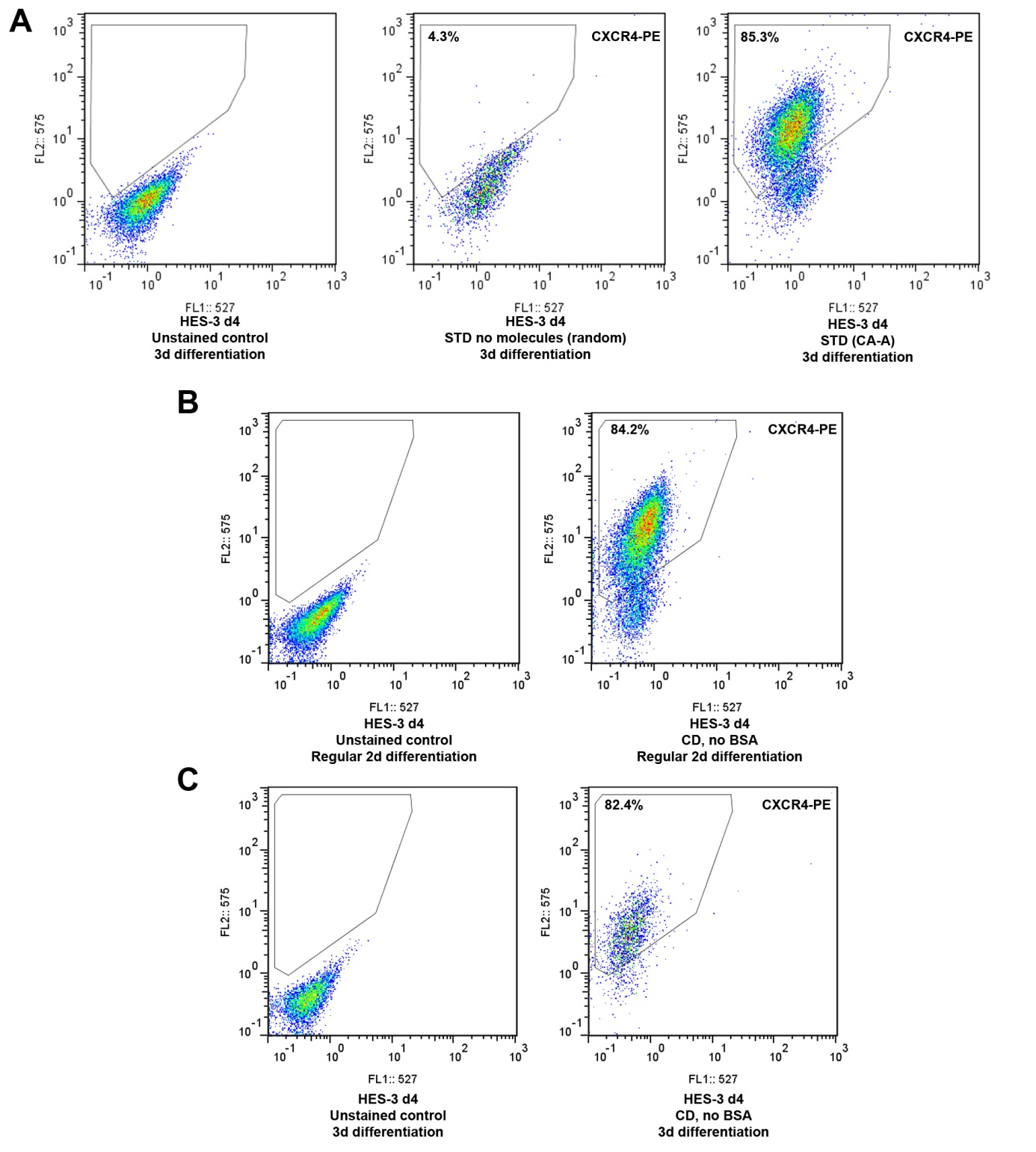


**Figure S7** **│** **Representative flow cytometry dot plots of standard (STD) and chemically defined (CD) differentiation in 2D and 3D into DE.** (**A**) From left to right: unstained control, CXCR4-PE staining of STD-3D differentiation w/o CHIR and activin A (random) and STD-3D differentiation. (**B**) CXCR4-PE staining of CD-2D differentiation. (**C**) CXCR4-PE staining of CD-3D differentiation.


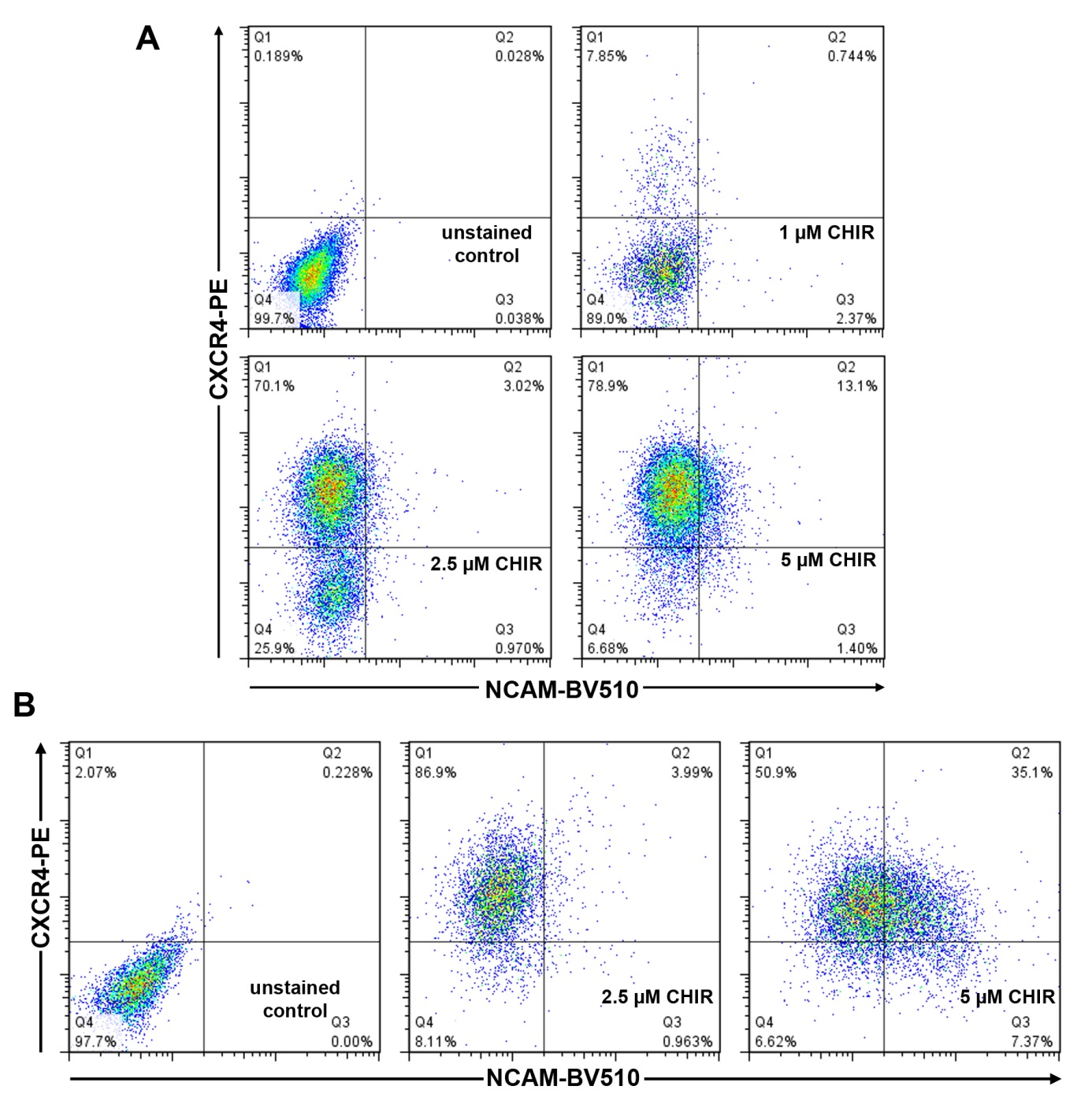


**Figure S8** **│** **Representative flow cytometry dot plots of xenogeneic-free (XF) differentiation in 2D and 3D into DE**. (**A**) CXCR4-PE and NCAM-BV510 staining of XF-2D differentiation with 1, 2.5 and 5 µM CHIR. (**B**) CXCR4-PE and NCAM-BV510 staining of XF-3D differentiation with 2.5 and 5 µM CHIR. Cell percentages are denoted in the quadrants. CHIR above 2.5 µM resulted in a substantial number of cells co-expressing CXCR4 and NCAM.

**Table S1 │** Low calcium buffer for Collagenase B.

| **Substance** | **Supplier** |
| --- | --- |
| 120 mM NaCl | Baker |
| 5.4 mM KCl | Marck |
| 5 mM MgSO_4_ | Merck |
| 5 mM Na-Pyruvate | Gibco |
| 20 mM Glucose | Sigma |
| 20 mM Taurin | Sigma |
| 10 mM Hepes | Sigma |
| 30 µM CaCl_2_ | Merck |

**Table S2 │** Primer pairs for gene expression analysis.

| **Gene Symbol** | **Primer Sequence 5’-3’** | **Exon spanning** | **Accession #** |
| --- | --- | --- | --- |
| *CXCR4* | Fw: tccaagctgtcacactccaa Rev: tgtagtaaggcagccaacagg | No | NM_001008540 |
| *FOXA2* | Fw: gggagcggtgaagatgga Rev: tcatgttgctcacggaggagta | Yes | NM_153675.2 |
| *G6PD* | Fw: aggccgtcaccaagaacattca Rev: cgatgatgcggttccagcctat | Yes | NM_000402 |
| *GSC* | Fw: gaggagaaagtggaggtctggtt Rev: ctctgatgaggaccgcttctg | Yes | NM_173849.2 |
| *HNF1B* | Fw: gaggaatgcaacagggcagaatg Rev: gaatgcctcctccttcctgcg | Yes | NM_000458 |
| *HNF6* | Fw: cgctccgcttagcagcatgc Rev: gtgtgttgcctctatccttcccatg | Yes | NM_004498 |
| *MIXL1* | Fw: ccgagtccaggatccaggta Rev: ctctgacgccgagacttgg | Yes | NM_031944.1 |
| *MNX1 (HLXB9)* | Fw: tccaccgcgggcatgatc  Rev: gcttgggccgcgacaggta | Yes | NM_005515 |
| *NANOG* | Fw: ccgagggcagacatcatcc  Rev: ccatccactgccacatcttct | Yes | NM_024864.2 |
| *NKX6.1* | Fw: ggcccggagtgatgcagagc  Rev: tcttcccgtctttgtccaac | Yes | NM_006168 |
| *PDX1* | Fw: cgttccagctgcctttcccat  Rev: ccgtgagatgtacttgttgaatagga | Yes | NM_000209 |
| *POU5F1* | Fw: cttgctgcagaagtgggtggagg  Rev: ctgcagtgtgggtttcgggca | Yes | NM_001173531.2 |
| *SOX2* | Fw: agctacagcatgatgcagga  Rev: ggtcatggagttgtactgca | Yes | NM_003106.3 |
| *SOX7 (v2)* | Fw: gatgctgggaaagtcgtggaagg Rev: tgcgcggccggtacttgtag | Yes | NM_031439.3 |
| *SOX9* | Fw: gcggaggaagtcggtgaagaacg  Rev: ggtcatggagttgtactgca | Yes | NM_000346 |
| *SOX17_Sybr* | Fw: gatgctgggcaagtcgtg  Rev: ctgcatgtgctgcacgc | Yes | NM_022454.3 |
| *TBP* | Fw: caacagcctgccaccttacgctc Rev: aggctgtggggtcagtccagtg | Yes | NM_003194 |
| *TUBA1A* | Fw: ggcagtgtttgtagacttggaaccc Rev: tgtgataagttgctcagggtggaag | Yes | NM_006009 |
